# The Promise of Optogenetics for Bioproduction: Dynamic Control Strategies and Scale-Up Instruments

**DOI:** 10.3390/bioengineering7040151

**Published:** 2020-11-24

**Authors:** Sylvain Pouzet, Alvaro Banderas, Matthias Le Bec, Thomas Lautier, Gilles Truan, Pascal Hersen

**Affiliations:** 1 Laboratoire Physico Chimie Curie, Institut Curie, PSL Research University, CNRS UMR168, 26 rue d’Ulm, 75005 Paris, France; alvaro.banderas@curie.fr (A.B.); matthias.lebec@curie.fr (M.L.B.); 2 Sorbonne Université, 75005 Paris, France; 3 Laboratoire MSC, UMR7057, Université Paris Diderot-CNRS, 75013 Paris, France; 4 Toulouse Biotechnology Institute, Université de Toulouse, CNRS, INRAE, INSA, 31400 Toulouse, France; thomas.lautier@insa-toulouse.fr (T.L.); gilles.truan@insa-toulouse.fr (G.T.); 5 Singapore Institute of Food and Biotechnology Innovation, Agency for Science Technology and Research, Singapore 138673, Singapore

**Keywords:** bioproduction, biomanufacturing, optogenetics, cybergenetics, dynamic regulation, bioprocess, biotechnology, photobioreactors

## Abstract

Progress in metabolic engineering and synthetic and systems biology has made bioproduction an increasingly attractive and competitive strategy for synthesizing biomolecules, recombinant proteins and biofuels from renewable feedstocks. Yet, due to poor productivity, it remains difficult to make a bioproduction process economically viable at large scale. Achieving dynamic control of cellular processes could lead to even better yields by balancing the two characteristic phases of bioproduction, namely, growth *versus* production, which lie at the heart of a trade-off that substantially impacts productivity. The versatility and controllability offered by light will be a key element in attaining the level of control desired. The popularity of light-mediated control is increasing, with an expanding repertoire of optogenetic systems for novel applications, and many optogenetic devices have been designed to test optogenetic strains at various culture scales for bioproduction objectives. In this review, we aim to highlight the most important advances in this direction. We discuss how optogenetics is currently applied to control metabolism in the context of bioproduction, describe the optogenetic instruments and devices used at the laboratory scale for strain development, and explore how current industrial-scale bioproduction processes could be adapted for optogenetics or could benefit from existing photobioreactor designs. We then draw attention to the steps that must be undertaken to further optimize the control of biological systems in order to take full advantage of the potential offered by microbial factories.

## 1. Merging Optogenetics and Bioproduction

### 1.1. Introduction to Bioproduction

Human societies have employed bioproduction since the ancient Egyptians first fermented grapes to produce ethanol for wine. Since then, bioproduction has been employed to address numerous global issues, such as the production of acetone using *Clostridium acetobutylicum* by Chaim Weizmann during World War One, the discovery of penicillin by Alexander Fleming in 1928 and the production of insulin by conventional *Saccharomyces cerevisiae* in the early 1980s [1]. Biomanufactured products have become ubiquitous components of our daily lives, including therapeutics (antibiotics, hormones [2], vaccines [3]), enzymes (stabilizers and cocktails [4]) and chemicals (amino acids, dyes [5], biodiesel [6]). The rise of systems biology (*omics* tools and databases, bioinformatics, metabolic engineering) and synthetic biology (cloning, metabolic and protein engineering, CRISPR, DNA synthesis) has expanded the possibilities for bioengineering, and sophisticated pathways have been successfully implemented into various cellular chassis; for example, the production of artemisinin [7], cannabinoids [8], and tropane alkaloids such as scopolamine [9]. Similarly to chemistry in the 19th century, biology is now shifting from a descriptive field to a constructive field, and bioproduction holds the potential to play a significant role by enabling the biomanufacturing of affordable medicines, and the sustainable production of high value-added chemicals and biofuels from renewable feedstock.

Despite these advances, biomanufacturing a new product remains challenging in many ways. Advances in systems and synthetic biology, as well as automation using high-throughput robotics, have reduced the time required to successfully produce a molecule or enzyme using a specific chassis. Nonetheless, achieving an economically viable and market-competitive production process using such whole-cell applications can be tricky, due to difficulties with scaling-up, the long duration of process development and the expensive, specific downstream processing steps. Therefore, extensive efforts have been made to optimize bioprocesses for existing engineered strains. The accumulation of the maximal number of producing cells is the first step towards maximizing production yield. However, the production of a molecule of interest will consume cellular resources and may generate toxic intermediaries or by-products. Thus, production often creates a stress or a burden that impairs cells’ ability to grow. Such burden can give rise to microbial heterogeneity and evolutionary escape, and lead to poor yields. Two-phase fermentation strategies are frequently implemented in bioreactors to minimize this burden. In the first phase, growth is favored; the production system is “silent” and cells actively divide without producing any heterologous component, allowing biomass to accumulate. In the second phase, production is “unleashed”, for example by inducing the expression of the recombinant enzyme or activating a synthetic pathway that leads to the production of the molecule of interest. During this second phase, the total content of metabolic precursors is divided between the cells’ endogenous needs and the synthetic pathway. Thus, the decoupling of growth from production—often irreversibly—has become standard in bioproduction, and this strategy is employed at every production scale. The switch from growth to the production phase can be mediated by various inducible promoters that respond to specific cues; for example, a triggered change in temperature [10] or pH [11], or the presence of a specific molecule such as IPTG (in *E. coli*), galactose (in *S. cerevisiae*) or methanol (in *Pichia pastoris*), or other changes in the environment (nutrient depletion, high cell density). Strong, non-reversible inductions are frequently employed; however, more comprehensive and subtle induction patterns are now increasingly preferred. In this context, the use of light as an inducer has attracted interest, given its ability to be finely tuned in space, time and intensity.

Optogenetics, i.e., using light to control cellular processes, is a versatile tool to induce production in industrial microorganisms. Light is a straight-forward output for computer control systems, as it is tunable down to the millisecond scale, reversible, and offers a range of different and compatible signals of various wavelengths. Moreover, light is more easily delivered and removed from bioreactors compared to the extensive media changes that would be required for chemical inducers, it is considered to be rather non-invasive to cells, and it is cheaper than chemical inducers. Only a small number of studies have applied this emerging strategy to bioproduction. However, researchers increasingly acknowledge optogenetics as a promising tool to achieve fine (and even real-time) control of complex biological systems. In this review, we aim to highlight recent advances and explore the limitations of merging optogenetics with bioproduction in the context of simple and more sophisticated bioproduction control strategies. We also discuss recent optogenetic instruments that will help to develop, characterize and control newly built strains, and the potential issues and opportunities that may be encountered during the scale-up of light-controlled bioproduction processes to the industrial scale.

### 1.2. Optogenetics

Light is widely used by biological systems, not only as an energy source, but also as a signal to which they respond in a variety of ways. Bacteria can express different types of photoreceptors to regulate, for example, the synthesis of protective pigments. Bacterial photoreceptors (opsins, LOV domains—blue light; CcaS/CcaR—red and green light) and plant cryptochromes (CRY2-CIB1—blue light), phytochromes (Phy-PIF—red/far-red light), or UV response systems (UVR8-COP1—UV light), form the basis of most optogenetic systems developed to date [12]. Although optogenetics was first used in neurosciences to excite or inhibit specific neurons via light-gated ion channels [13], the technique has recently been extended to other mammalian cell types to study developmental timing and coordination [14], regulatory cascades’ responses to dynamic signals [15], and cellular biophysical processes [16]. With respect to microbial systems, numerous optogenetic systems have been developed and used to investigate the protein control of biofilm formation, metabolic flux control (reviewed in [17]) and dynamic regulation of gene expression to dissect pathway dynamics [18]. In non-neural studies, light is used to control protein interactions, which can give rise to various molecular functions (dimerization, relocalization, anchoring, phosphorylation/activation, oligomerization). In the context of bioproduction, it is the transcriptional control resulting from such optogenetic interactions that is mostly employed.

**Figure 1:**
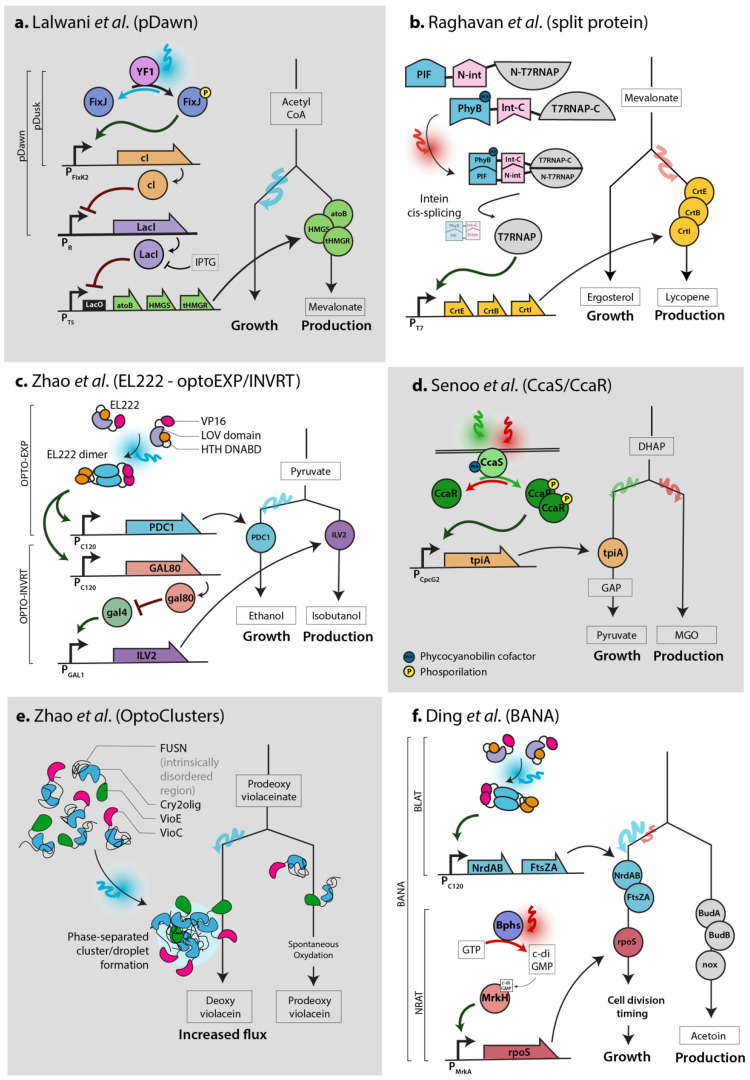
Different optogenetic systems can be used to achieve flux control in various ways. Sketch of circuits adapted from each paper. (**a**). Lalwani et al. [26] controlled mevalonate production using the pDawn system. (**b**). Raghavan et al. [28] used a light-responsive split T7 RNA polymerase (T7RNAP) to control lycopene production. (**c**). Zhao et al. [32] used EL222—composed of the VP16 trans-activation domain, light-voltage photosensitive domain (LOV) and helix-turn-helix DNA-binding domain (HTH DNABD)—to dynamically regulate isobutanol production. Blue light stimulation activates gene expression in the OPTO-EXP system, and gene expression in the optoINVRT system is activated in the dark via the GAL regulatory pathway (GAL4 is constitutively expressed in this system). (**d**)**.** Senoo et al. [38] used the CcaS/CcaR system to regulate glucolysis flux. (**e**). Zhao et al. [40] developed a light-induced phase-separated cluster formation. Sequential enzymatic reactions are favored in this conformation. (**f**). Ding et al. [41] used the EL222 and Bphs systems to regulate division timing in *E. coli* to restore the growth rate and improve acetoin production.

Some optogenetic systems are particularly efficient and versatile. In the pDusk system [19], the histidine kinase YF1 phosphorylates the transcription factor FixJ in the dark, which activates transcription from the *FixK2* promoter. This process is reversed by blue light stimulation. In contrast, to achieve induction upon blue light stimulation, the pDawn system [19] (Figure 1a) was built by adding another regulation step: by placing the lambda phage repressor cI under the control of the *FixK2* promoter, the repressor cI is repressed by light, which enables the activation of the target promoter *pR*. In the PhyB-PIF system [20], the PhyB and PIF proteins dimerize upon red light stimulation (Figure 1b) and dissociate when exposed to far red light. The two photosensitive domains PhyB and PIF are usually fused separately to effector protein domains, typically a DNA-binding domain and a trans-activation domain, to regulate transcription. This interaction requires the presence of the cofactor phycocyanobilin (PCB), which is naturally present in plants, but must be externally added or engineered in microbial systems. In the single-component EL222 system [21] (Figure 1c,f), the engineered EL222 protein (composed of a caged DNA-binding domain, LOV domain and VP16 transactivation domain) homodimerizes upon blue light stimulation, which promotes DNA binding and transcription from the C120 promoter. When the CcaS/CcaR system is stimulated by green light [22] (Figure 1d) in the presence of the cofactor PCB, membrane-bound CcaS phosphorylates the transcription factor CcaR, which activates transcription from the *Cpcg2* promoter. This process is reversed by red light, which therefore prevents transcription. Finally, similar to the PhyB-PIF system, the Cry2 and Cib1 proteins of the CRY2-CIB1 system [23] dimerize upon blue light stimulation (which is reversed in the dark) due to the interaction between photons and the (naturally present) protein cofactor flavin adenine dinucleotide (FAD). Cry2 has also been engineered to self-multimerize upon light stimulation [24], as illustrated in Figure 1e. For more details on these and other optogenetic systems, we recommend consulting optobase.org [25].

### 1.3. Adapting Induction Systems for Optogenetics

In an effort to adapt current genetic induction systems for bioproduction, several systems have been designed to facilitate the transition of existing industrial organisms from chemical to optogenetic induction without the need for full reconstruction or redesign.

Optogenetic regulation has been achieved in *E. coli* by combining optogenetics with classical IPTG, arabinose or T7 regulation systems. IPTG, the gold standard inducer in *E. coli*, binds the LacI repressor and thus induces the expression of genes containing a *lac* operator in their promoter by preventing LacI from shielding DNA from RNA polymerase. Lalwani et al. [26] (Figure 1a) placed *LacI* under the control of the pDawn system, so that blue light induces *LacI* expression and therefore represses the genes of interest. In contrast, the absence of light represses *LacI* expression and therefore activates the various IPTG-inducible promoters. This system was optimized to reduce leakiness, and although the expression dynamics are slower than those of IPTG induction systems (2 h delay), the final induction levels are higher and production exceeds that of the IPTG induction systems. Thus, the pDawn system is a successful alternative to IPTG induction, and has already been tested and applied to bioproduction and scaled-up to 2 L [26]. The pDusk and pDawn systems also highlight the possibility of activating or repressing a system by illumination or darkness, depending on how the optogenetic system is connected to the bioproduction system. Similarly, Romano et al. [27] substituted arabinose with light to control the BAD promoter by switching the endogenous dimerization domain of the AraC transcription factor with the VVD blue light optogenetic domains, which dimerize upon blue light stimulation. Thus, this system is compatible with pBAD-based vectors or strains, which are frequently used in smaller-scale studies. Finally, expression control using the T7 promoter is another standard in *E. coli* and is also used in *S. cerevisiae*. Raghavan et al. [28] used a split version of the T7 RNA polymerase (T7RNAP), with each part of split-T7RNAP fused to the N-or C-terminus domain of an intein, and also to either the Phy or PIF component of *Arabidopsis thaliana* phytochrome (see Figure 1b). The red light illumination of the PCB cofactor triggers the Phy-PIF interaction, which allows the intein domains to interact; trans-splicing occurs to deliver a functional T7RNAP that promotes the expression of genes under the control of the *T7* promoter. Raghavan et al. successfully used this system to control the production of lycopene in *E. coli*. However, this system relies on the PCB cofactor and is not reversible, since T7RNAP is stabilized once trans-spliced. A similar strategy was used to increase the controllability and simplicity of T7RNAP. Baumschlager et al. [29] created a split version of T7RNAP that heterodimerizes upon blue light illumination due to the presence of engineered VVD domains fused to each T7RNAP termini (“Magnet” domains [30]), to create an Opto-T7RNAP system that exhibits rapid, reversible dynamics. Another version, paT7P-1, was developed by Han et al. [31].

Zhao et al. [32] connected the well-studied galactose regulation system used in the yeast *Saccharomyces cerevisiae* to the EL222 optogenetic system (Figure 1c). The authors first built the simple OPTO-EXP system, in which the protein EL222 induces the transcription of genes controlled by the C120 promoter (more subtle versions of this promoter have since been made and evaluated [33]). Then, to reverse the system and achieve activation in the dark (OPTO-INVRT), Zhao et al. placed *GAL80* under the control of the C120 promoter. In the presence of the (non-naturally) constitutively expressed GAL4 transcription factor, genes under the control of the GAL promoter are expressed in the dark. Upon blue light illumination, GAL80 is expressed and inhibits the activity of GAL4, therefore repressing genes under the control of the GAL promoter. Using both the OPTO-EXP and OPTO-INVRT systems, genes can be actively induced or repressed in a mutually exclusive way given the presence or absence of light, making this bidirectional system particularly versatile. Zhao et al. used this system to achieve dynamic control of isobutanol production up to the 2 L scale at high cell density.

It is worth noting that optogenetic systems have also been implemented in non-conventional microorganisms, such as *Pseudomonas putida* [34], and other chassis already used in industry, such as *Bacillus subtilis* [35]. The widely used yeast *Pichia pastoris* has not, to date, been adapted to optogenetic control, but we expect this to be achieved within a few years. Moreover, widely used synthetic biology systems have also been adapted for optogenetics: photo-inducible CRE recombinases [36] and photosensitive degrons [37] could be used to complement the current optogenetic systems used for bioproduction.

## 2. Control Strategies

### 2.1. Simple Switch for Flux Rewiring

Flux control lies at the heart of bioproduction strategies. To prevent production from impairing the accumulation of biomass, production is inhibited and induced after a growth phase; only then is metabolic flux redirected towards the product of interest. Chemicals or auto-induction systems have been extensively used to achieve flux control, and optogenetics has the potential to perform at least as well as other methods of induction, while also improving controllability. Using the CcaS/CcaR optogenetic system, Senoo et al. [38] (Figure 1d) and Tandar et al. [39] controlled the expression of the *tpiA* and *pgi* genes, respectively, two important genes that channel metabolite flux towards glycolysis in *E. coli*. Both studies demonstrated enrichment in their respective competing pathways, as expected. To obtain more insight into induction timing, Raghavan et al. [28] used the PhyB-PIF system (Figure 1b) and Lalwani et al. [26] used FixJ (Figure 1a) to explore light induction at different optical densities during growth. Raghavan et al. found that the illumination pulse was most efficient during the late exponential phase of growth, and Lalwani et al. found that constant illumination at an OD of about 1 was optimal. Thus, similarly to chemical inducers, the timing of illumination must be considered in the context of the growth state; the optimal timing may essentially depend on the induction time delay of the optogenetic system, as well as the amount of burden that the cells will experience.

Compartmentalization is another strategy that can be controlled using optogenetics to redirect flux towards a specific metabolite, by using higher-order structures that bring enzymes close to each other to create reversible metabolons inside the cell. Thus, intermediate metabolites are channeled to the next enzymes in the pathway, located in close proximity, which increases the final product yield. Zhao et al. [40] built two systems for this purpose: OptoClusters (Figure 1e) is based on the engineered Cry2olig domain [24], which oligomerizes upon blue light stimulation, fused to the intrinsically disordered region (IDR) FUSN to create a phase-separated synthetic organelle. On the other hand, the PixELLs system (based on PixE/PixD from *Synechocystis* sp. fused to FUSN) loses its phase-separated structure upon blue light stimulation. These two systems form or dissociate droplets within seconds upon light stimulation. After optimization, Zhao et al. demonstrated flux redirection control using the VioC and VioE enzymes fused to the optogenetic components to control deoxyviolacein formation (Figure 1e).

Although light can be used as a simple switch-like inducer, it is its reversibility and high-controllability that makes it a singular tool facilitating the fine-tuning of cell-processes in a dynamic way.

### 2.2. Dynamic Switch

**Figure 2:**
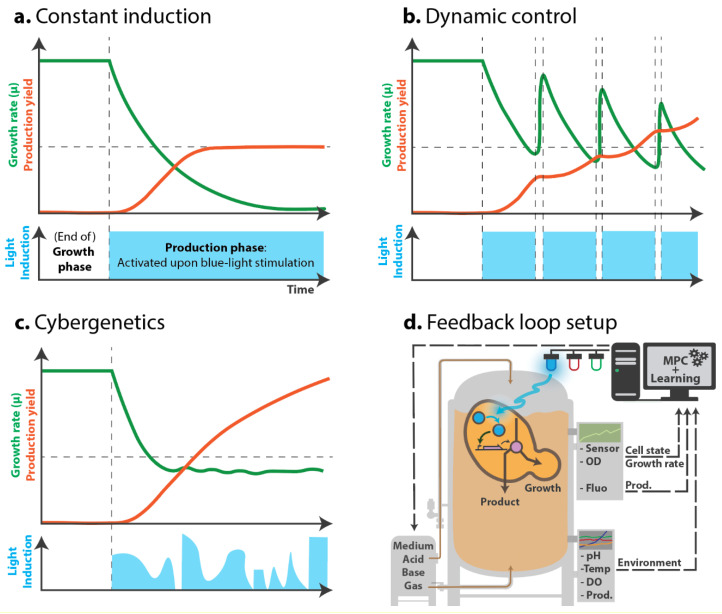
Sketch of different putative light-induction patterns to balance growth and production. (**a**–**c**). Green curve: growth rate; orange curve: production yield. In blue: light induction pattern that activates bioproduction. (**a**). With a constant and irreversible induction, production yield increases and plateaus as growth plummets and stalls due to bioproduction-induced stress. (**b**). Dynamic control of induction: alternating growth and production phases allows cells to “recover” from the production phase and resume growth, before starting to produce again. This strategy was successfully implemented in Zhao et al. [32]. (**c**). Cybergenetic control would allow for balancing, in real-time, growth and production, by inducing production given the cell’s state. In this sketch, keeping cell growth at a certain rate could ensure a low stress level that would result in a sustained productivity. (**d**). To implement the feedback loop, besides regular bioproduction parameters control (medium composition, pH, temperature, etc.), the optogenetic actuator (induction upon blue light in this example) is regulated given an output from the system (a cue from the environment, a measure of the growth, or cell fluorescence indicating production level, stress or metabolite pool status) that is interpreted by an algorithm (Model predictive control - MPC) to predict and act upon the behavior of the cell and balance the bioproduction process.

Many recent papers mention the possibility of using optogenetic systems to achieve dynamic control over bioproduction. During the production phase, cells may still undergo some growth (or simply maintenance) and experience a burden. However, this trade-off between growth and production can be more closely controlled (Figure 2). This idea was confirmed by studies that used stress-related promoters to modulate the induction of bioproduction systems [42] and lead to increased production. This “host aware” [43] or “burden-driven” strategy shows that dynamic control must be considered, based on the cellular state of the producing cell, in order to improve yields. 

Given the ease with which such strategies can be implemented using optogenetics, dynamic control is starting to appear in bioproduction studies. For instance, Lalwani et al. [26] tested the ability of illumination duty cycles to control protein expression levels over a period of about 17 min. They managed to recapitulate a full range of induction strengths (similarly to [33]), which can be very difficult to obtain using chemical inducers (such as IPTG) that induce high expression levels regardless of their concentration, i.e., in a more switch-like manner. Controllability is significant, since strong and sudden induction is not necessarily the best strategy to maximize yield. Indeed, an overload of toxic intermediates and overexpression of a recombinant protein may impair folding and create stress [44], and therefore directly limit production.

Dynamic induction could enable repeated and reversible switching between the growth and production phases, which would let cells produce, then “recover” from production, and then produce again later. Current chemical induction systems may allow such dynamic control to an extent, but auto-induced systems (based on nutrient limitation or cell density) are frequently irreversible. Indeed, using chemical inducers such as IPTG would require complex media changes, and inducers such as galactose or methanol are metabolized by the cells, and thus hard to control. Temperature-sensitive promoters could act as reversible systems, but provide low reactivity. In addition, temperature changes involve hard-to-handle bioprocesses and certain temperatures will not necessarily fit the thermal optima of the enzymes required for endogenous and synthetic pathways. Using optogenetics to control the production of isobutanol in *S. cerevisiae*, Zhao et al. [32] applied bidirectional control using the OPTO-EXP and OPTO-INVRT systems to express *PDC1* (essential for fermentation and growth) only upon light stimulation, and express isobutanol-related genes in the dark. This way, using light, they not only favored the channeling of metabolic flux towards production, but also blocked the competing route; instead of just opening a single valve, they opened one and closed another to specifically control growth and production. Using this system as a simple switch, Zhao et al. realized that the cells were unable to consume all of the glucose in the medium by the end of the production phase, probably due to metabolic arrest related to NAD^+^ depletion. However, using periodic 30 min light pulses to activate PDC1 every 10 h, the NAD^+^ pool could be restored; this method tripled the amount of isobutanol produced. Most importantly, the authors demonstrated that dynamically controlling growth and production—not simply just separating them—has substantial potential in improving yield (Figure 2b). 

In light of these advances, the next logical step is to best adjust the induction pattern based on the cell’s state or content of specific metabolite pools, and automatize this dynamic control in real-time. Such control could be achieved using a cybergenetics approach.

### 2.3. Cybergenetics

Cybergenetics seeks to combine control engineering with synthetic biology as a mean to control biological processes in real-time from outside the cell. Cybergenetics requires three elements: an actuator (for example, an optogenetic system), a biosensor (a reporter of the metabolic state, via a fluorescent protein level or growth rate) and a computer algorithm to control the actuator (via light) based on the biosensor output (subtly reviewed by Carrasco-Lopez et al. [45]). While simple dynamic induction is considered open-loop control (Figure 2b), cybergenetics aims to close this loop to achieve automation via real-time feedback loop control (Figure 2c,d). Such closed-loop control is especially important when experiments yield poor reproducibility, as closed-loop control can adapt and stabilize noisy systems.

Cells naturally use internal control mechanisms to adapt to changes in their environment, cope with fluctuations in internal metabolite pools and respond to stress. In the context of bioproduction, this natural ability has already been exploited to balance growth and production. Taking advantage of the innate regulatory networks of *E. coli*, Ceroni et al. [42] used the stress-responsive pHtpG1 promoter to control the expression of a guide RNA to repress—via a constitutively expressed dead Cas9—the expression of a heterologous gene used to produce the fusion protein VioB-mCherry. Using this “burden-responsive biomolecular feedback controller”, they managed to improve production using a continuous production strategy, but did not compare the results to the two-phase strategy. Such a host-aware approach has not yet been implemented using optogenetics, though this step appears feasible and promising. Another strategy to monitor burden in the cell employs metabolite-responsive transcription factors (MRTFs; see [46]) to report the level of a key metabolite pool required for the production of the final product, or the final product itself, to prevent stress.

Automated control of protein expression levels is also of particular interest. Indeed, as mentioned before, excessive expression can decrease production. Given the potential changes in the environment and cell density, closed-loop control is required to maintain constant per-cell expression throughout the production phase. Using fluorescent proteins as biosensors, such real-time control was demonstrated using chemicals as inducers [47,48,49] and using optogenetics with the Phy-PIF [50] and CcaS/CcaR systems [51].

In addition to controlling the intracellular concentration of a protein, Milias-Argeitis et al. [51] showed that the growth rate of *E. coli* could be regulated via optogenetics using the CcaS/CcaR system to control the *metE* gene, which is responsible for the last step of methionine biosynthesis. Controlling the growth rate in bioproduction is important, since productivity can be either proportional or inversely proportional to growth, or only be optimal at a certain growth rate [52]. In this context, by tuning the intensity and time of both blue and near-IR illumination, Ding et al. [41] finely controlled the division timing of *E. coli* by connecting their custom optogenetic systems to control the expression of the ribonucleotide reductases *NrdAB* or *NrdA* and division proteins *ftsZA* or *SulA*, which influence dNTP biosynthesis and cell division (Figure 1f). This system enhanced the yields of acetoin and poly(lactate-co-3-hydroxybutyrate) by shortening and prolonging cell division, respectively (which restored a reduced growth rate in both cases), in two different strains, up to the 5 L scale. Although closed-loop feedback control was not employed, this method demonstrates the potential of growth control for bioproduction and the possibility of combining several optogenetic systems responding to different wavelengths. Both aspects could very well be applied in the context of cybergenetics (Figure 2d).

## 3. Scale-Up Instruments

### 3.1. Milliliter Scale

Optogenetic experiments with microbial cultures require dedicated equipment to screen for and characterize strains, and to initiate the scale-up work. This is why, at present, most labs either develop new devices in-house or adapt previously published systems, mostly in a highly flexible Do-It-Yourself (DIY) spirit. Therefore, basic knowledge of electronics, a 3D printer, a laser cutter and some device programing, i.e., the presence of a typical fablab, are usually required. The resulting device should be robust, not too expensive, and rather simple to build and calibrate.

**Figure 3:**
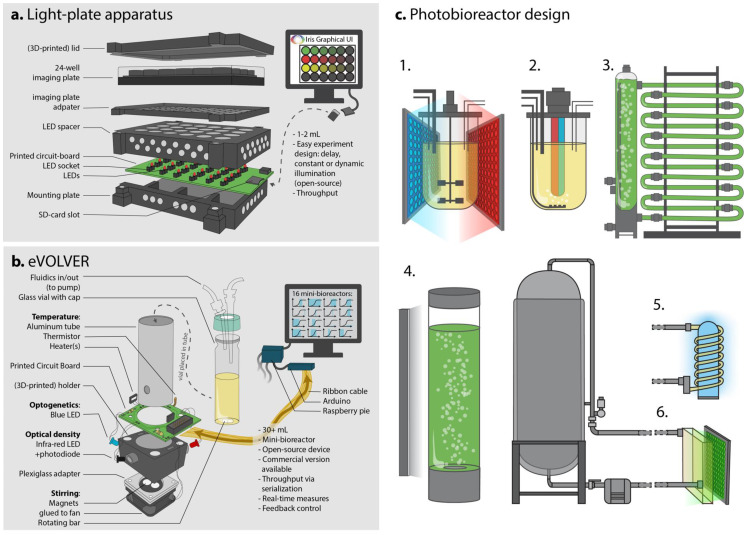
Instrumentation for optogenetic control and scale-up of bioproduction. (**a**). The light-plate apparatus [53] allows screening and simple experiments using small culture volumes in imaging plates and easily-programmable well-independent experiments. (**b**). The eVOLVER system [57] used as mini-bioreactors allows larger scale (30 mL) cultures and dynamic real-time control, as well as culture and illumination conditions screening thanks to its 16-unit array. (**c**). Bioreactor design to improve light penetration is crucial to successfully implement optogenetics as an induction method at the industrial scale. (**1**). Externally illuminated 10 L fermenter-type bioreactor using LED panels. Such systems offer the best control over every process parameter. (**2**). Internally illuminated 10 L bioreactor, which would allow better light penetration in a dense culture medium. Note the subsequent change in the gas-exchange strategy. (**3**). Stacked tubular photobioreactor, typically used for algal culture, increases the surface to volume ratio of large culture volumes to maximize light exposure. Up to thousands of liters of cultures can be handled, but temperature may be difficult to control and O_2_ and CO_2_ levels may fluctuate [61]. (**4**). Standard 250 L bubble column algal photobioreactor (illumination pane on the left) is considered an intermediate volume for algal cultivation. It is easy and cheap to set up and can be scaled-up, but has a relatively low surface to volume ratio. (**6**). Connection of an illumination chamber to a 1000 L bioreactor could allow illumination of flowing cells and reduce the need for large fermenter-type bioreactor redesign. Similarly, in (**5**), a loop passing out of the fermenter coils around a light source to enhance exposure to light.

The Light-Plate Apparatus devised by Gerhardt et al. [53] (Figure 3a) was one of the first platforms able to accommodate 24-well plates and apply two wavelengths per well. It only requires printed circuit boards (PCB) that can be easily ordered from specialized companies, some LEDs, LED sockets, 3D-printed parts for assembly, a soldering iron, a chip burner and a few screws. With this system, once the illumination power of the LEDs has been calibrated, the programing is very simple thanks to the graphical user interface (GUI) provided and experiments can be designed fairly quickly, enabling various illumination intensities and patterns to be independently delivered to any well, providing a good throughput of strain testing. Similar systems have been reported for microwell plates (up to 96-wells or more) [27,54] or larger volumes (up to 10 mL [55,56]). These types of systems are great for small-scale experiments, such as strain characterization and screening various illumination patterns or media compositions. However, reading an output (a fluorescence level, a growth rate, etc.) from each well can be hard to automate and labor-intensive, especially if time-course profiles have to be achieved manually. For this type of study, and at such scale, plate-readers may be of value, particularly if the plate-reader can illuminate various wells independently while simultaneously measuring fluorescence or the optical density. Such sophistication is already available in state-of-the-art plate-readers. Yet, such instruments remain much more expensive and harder to handle than DIY devices.

### 3.2. Mini-Bioreactor Scale and Feedback Implementation

Once a producing optogenetic strain has passed screening and milliliter small scale characterization, it can be time to monitor its growth and production dynamics at larger scale and test illumination patterns accordingly. DIY mini-bioreactor systems have recently been developed by different labs to enable such real-time measurements and, most importantly, contain illumination setups. These systems include the eVOLVER [57] (Figure 3b) and Chi.Bio [58] systems, both of which work with at least 30 mL culture volumes, are customizable, and allow fluidic inputs and illumination at various wavelengths, as well as optical density measurements, and stirring and temperature control. Both devices are open-source projects, freely providing all details regarding the construction steps and components used. Both of the authors also offer commercial versions of their devices with appropriate GUIs for designing experiments and extracting output data. Usually, a set of these small bioreactors (typically 16 mini-bioreactors) are used to screen various media compositions, illumination patterns, temperatures, stirring speeds, etc. Moreover, compared to previously discussed well plates-based optogenetic instruments, eVOLVER and Chi.Bio enable the manipulation of larger culture volumes, an important intermediate step towards scaling-up to industrial conditions. Therefore, these systems combine the advantages of a small, versatile screening tool, while actually more closely mimicking larger-scale settings in terms of control and monitoring. Note, however, that pH and dissolved oxygen levels are two important factors that are not considered in those devices—although they could be implemented in the future. The embedded optogenetic hardware (mainly LEDs) can be easily tuned, and Chi.Bio especially allows for the measurement of at least two fluorescence outputs thanks to its seven-color LED and small spectrophotometer. To better control the production given the cell’s state, the devices’ measurement capacities will come to be crucial if fluorescent biosensors are to be used to establish automatized real-time control.

Since these devices are quite small and emit relatively powerful light, there are only a few concerns related to poor light penetration or distribution in the medium. However, it will be crucial to address this issue when scaling-up to industrial settings. Although the small size of those instruments comes as an advantage in the lab, variations in growth and production caused by the effects of larger culture volumes cannot be assessed using these devices, such that they will not replace pilot-scale testing. Nonetheless, they will be essential to start balancing growth and production and fine tune computer control models.

### 3.3. Industrial Settings and Photobioreactors

Even in the absence of optogenetics, scaling-up to the industrial scale (from 10 to 10,000 L) represents a challenge for any potential industrial strain. At such scales, the raw materials used for fermentation will change given the costs and quantities of chemicals required to piece together the culture medium. Moreover, mixing and oxygenation become more demanding due to the energy needed to move dense culture volumes; the inoculum volume may become critical, and pressure and shear stress may be unevenly distributed. All of these changes might impact the performance of the strain at the larger scale [59], which may prompt researchers to another round of refinement of the initial strain design. One of the main concerns regarding optogenetics at larger scales is the penetration of light in the medium. Indeed, high-density cell cultures can become very opaque, which would prevent light from reaching a maximum number of cells (light-shading effect), or make it hard to sufficiently activate the biological process (due to dilution of the signal). Simply adding light panels around a standard bioreactor (Figure 3(c1,c4)) can overcome these issues at relatively small scales (2 to 250 L), but poses serious issues for scaling-up. Indeed, the low surface to volume ratio of larger bioreactors is a crucial factor that must be considered in the context of light exposure [60].

Light penetration is a crucial issue for the cultivation of micro-algae and cyanobacteria. As these organisms are also a valuable chassis for bioproduction (mostly for biofuels), this issue has actually been addressed in various ways, along with how to deal with pH, dissolved oxygen (DO), temperature and mixing. Different types of photobioreactors are commercially available and used to produce various biomasses or specific compounds [60]. Closed-type algal photobioreactors, such as airlift or bubble columns (Figure 3(c4)), stacked tubular (Figure 3(c3)), flat-plate and multilayered photobioreactors [61], are illuminated from the outside. These designs aim to increase the surface area of the culture in contact with, mostly, ambient light or custom light sources, and counteract the shading effect caused by high culture density or large volumes. In bioproduction, yeast cultures can result in an extremely dense, very opaque, paste-like textured medium, so that the number of cells is maximized to reach higher titers. The compatibility of algal photobioreactors with such high-density yeast cultures remains to be tested; and although light penetration is the main advantage of such photobioreactors, large-scale algal photobioreactors may not be adaptable to heavy and dense cultures (harder to pump, harder to cool down, less diffusion in the medium). Besides, algal photobioreactors often rely on continuous cultures, which generates a significant risk of evolutionary escape in burdened yeast cultures. We suggest that lower-density yeast cultures may potentially benefit light penetration and controllability. Owing to a refined dynamic control strategy, the reduction in the final biomass could be compensated for by increased production per cell; the trade-off between these aspects will need to be balanced.

Another solution to the light penetration issue could be the use of “inverted” optogenetic systems, where genes for the production phase are activated in the dark, when light becomes scarce, i.e., when a certain cell density is reached [26]; however, this would hardly allow for dynamic control.

Finally, it may be possible to redesign or tune existing large-scale bioreactors. Most standard large-scale bioreactors are made of metal, which limits the use of light; however, internal illumination may overcome this issue (Figure 3(c2)). Internal illumination is usually achieved using optical fibers or light wells that reach inside the culture medium and transmit light collected outside into the bioreactor, or by using fluorescent lamps. LEDs are increasingly being used, as they offer the advantages of being able control the exact wavelength used in the medium [60] and even possibly deliver several wavelengths to control combinations of multiple optogenetic systems. However, such modifications may also conflict with other aspects of the bioreactor, such as the stirring mechanism (Figure 3(c2)). As an alternative to modifying the original design, one could also take advantage of existing plug devices—such as probes or external loops—which may be used to easily implement optogenetic control into existing bioreactors. For instance, during the synthesis of organic chemicals, photochemical reactors sometimes use external illumination chambers (flat panels or coils around a light source; see Figure 3(c5,c6)). Illumination chambers could enable the regulation of the optogenetic system based on the regulated flow of cells across the illuminated chamber, therefore tuning the amount of light received per cell per time. Such an approach may minimize the redesign of the bioreactors and thus may be a more feasible and economically advantageous first step to combine optogenetics and bioproduction at a large scale.

Finally, it is worth noting that modeling light distribution given bioreactor design [62], together with the optogenetic system, metabolic pathways and the bioprocess, will be key to optimizing bioproduction [63,64].

## 4. Discussion and Conclusions

As metabolic engineering increasingly relies on the fine tuning of intricate endogenous and synthetic pathways, light is becoming the inducer of choice for bioproduction—as evidenced by the fact that many standard induction systems have already been connected to optogenetic systems. Light can be used to regulate metabolic valves in a similar manner to existing inducers used in bioproduction: by unleashing bioproduction after a growth phase. However, light can also be used to control cellular growth itself by regulating endogenous essential genes, or even used to actively and dynamically balance both bioproduction and growth using bidirectional optogenetic systems. Optogenetics enables increasingly simpler and more precise dynamic control over these processes, and holds the promise of maximizing yields from industrial organisms engineered for complex pathways that frequently require multilevel regulation. The automation of this dynamic control via a cybergenetics approach seems the natural next step: under industrial conditions, every aspect of the culture is rigorously monitored and controlled, including temperature, pressure, pH, medium composition, dissolved oxygen and optical density. In addition to controlling these external cellular cues, cybergenetics aims to control the intricate internal behavior of cells based on a designated output (Figure 2d). This output can be the level of the final product detected using a biosensor, the cellular concentration of an enzyme or component of the pathway, or the burden that production represents—which can be detected using metabolite pool biosensors, stress-related promoters or directly monitoring the growth rate. Cybergenetic control could be achieved using various optogenetic systems, i.e., actuators, and biosensors that currently exist (with many others in development) [65]. The last element needed for cybergenetics is an appropriate control algorithm that takes into account and predicts the behavior of the cell, as well as how the culture density affects the diffusion of light—to better control the bioproduction *versus* growth trade-off. Various algorithms have been tested for the control of rather simple behaviors, such as the expression level of a fluorescent protein, and some of these already use optogenetic systems. Building models to represent burden will require extensive experimental fitting, and this may be facilitated by implementing machine learning strategies that can train from concrete experimental data [66]. Such models can easily be tested on smaller scales in the lab before being adapted to large culture volumes.

Many optogenetic devices have been developed in recent years to tackle every step of strain development; the real challenge now is to prove that optogenetics can truly be used in the context of bioproduction at the industrial scale in an economically sustainable manner. Thanks to their generally well-developed graphical user interfaces, small-scale devices that allow illumination of imaging plates will be convenient for the throughput testing of various media compositions, genetic designs and, most importantly, light illumination intensities or basic illumination patterns. Mini-bioreactors will enable the testing of larger volumes and fluidics inputs and outputs. They offer the capacity to control temperature and stirring and, crucially, to monitor growth and production in real-time—as well as implement feedback loop control based on such outputs. Mini-bioreactors that enable the use of optogenetics without critical light penetration issues will allow users to start to tune the dynamic control of the strain to optimize bioproduction. However, due to the opacity of high-density yeast cultures, light penetration becomes one major issue when shifting dynamic control from the small scale to the industrial scale. Although light panels or internal illumination strategies may be suitable solutions for bioreactors up to about 250 L, larger scale bioreactors will require further modeling, redesign or tuning, though existing algal photobioreactors or photochemistry may provide inspiration.

The use of light as an inducer can facilitate real-time dynamic control strategies. The first applications of light to bioproduction—as well as the optogenetic instruments presented here—show that although challenges remain to be solved, the application of optogenetics to bioproduction holds the promise of maximizing yields in bioproduction; promise that can be expected to be fulfilled in the years to come.
